# Vertical Movement of Head, Withers, and Pelvis of High-Level Dressage Horses Trotting in Hand vs. Being Ridden

**DOI:** 10.3390/ani15020241

**Published:** 2025-01-16

**Authors:** Hilary M. Clayton, Sarah Jane Hobbs, Marie Rhodin, Elin Hernlund, Mick Peterson, Rosalie Bos, Filipe Serra Bragança

**Affiliations:** 1 Large Animal Clinical Sciences, Michigan State University, 736 Wilson Road, East Lansing, MI 48824, USA; 2 Research Centre for Applied Sport, Physical Activity and Performance, University of Central Lancashire, Preston PR1 2HE, UK; sjhobbs1@uclan.ac.uk; 3 Department of Anatomy Physiology and Biochemistry, Swedish University of Agricultural Sciences, S-750 07 Uppsala, Sweden; marie.rhodin@slu.se (M.R.); elin.hernlund@slu.se (E.H.); 4 Biosystems and Agricultural Engineering and UK Ag Equine Programs, University of Kentucky, Lexington, KY 40546, USA; mick.peterson@uky.edu; 5 Sporthorse Medical Diagnostic Centre, SMDC, Hooge Wijststraat 7, NL-5384 RC Heesch, The Netherlands; bos@sporthorsemdc.co; 6 Department of Clinical Sciences, Faculty of Veterinary Medicine, Utrecht University, Yalelaan 112-114, NL-3584 CM Utrecht, The Netherlands; filipe.braganca@slu.se

**Keywords:** head asymmetry, withers asymmetry, pelvic asymmetry, limb pro-/retraction, collected trot, extended trot, fit to compete

## Abstract

Prior to international competitions, dressage horses are evaluated for fitness to compete while trotting in hand on a firm surface. This study compares the kinematics of experienced dressage horses trotting under fitness-to-compete conditions vs. performing collected and extended trot when ridden on a sand-fiber arena surface. The hypotheses are that the vertical range of motion (ROM) and left–right asymmetries in minimal and maximal heights of axial body segments at ridden trot exceed those when trotting in hand. Inertial measurement units (IMUs) attached mid-dorsally to the head, withers, and pelvis of 19 actively competing dressage horses measured the vertical ROM and left–right asymmetries in minimal (MinDiff) and maximal (MaxDiff) heights of the midline sensors. The vertical ROM was greater for both types of ridden trot, reflecting greater impulsion in response to the riders’ aids. Head MinDiff/MaxDiff and withers MaxDiff were significantly higher under both ridden conditions. Pelvis MinDiff was significantly the largest for collected trot. Compared with trot in hand, left–right differences in limb protraction were larger for extended and collected trot in the forelimbs but only for extended trot in the hind limbs. The rider’s influence increases the horse’s impulsion and vertical ROM, which may exacerbate inherent asymmetries in muscular strength when lowering the haunches and elevating the withers.

## Simple Summary

Before entering high-level dressage competitions, horses are inspected for lameness while trotting in hand, but it is unclear how motion asymmetries change when horses are ridden. This study measures axial and limb asymmetries to test the hypothesis that ridden horses have greater vertical movement asymmetry of the head, withers, and pelvis than when trotting in hand. Nineteen dressage horses were evaluated trotting in hand on a firm surface and being ridden by their trainer in an arena with sand-fiber footing at collected and extended trot. Inertial measurement units (IMUs) on the head, withers, and pelvis measured data describing vertical motion and left–right asymmetry under the three trotting conditions. IMUs on the cannon bones measured left–right symmetry in limb pro-/retraction. Ridden horses had larger vertical ranges of motion of the head, withers, and pelvis, which were ascribed to the riders’ effects on impulsion and engagement. Ridden horses had larger asymmetries in head and withers MaxDiff and pelvic MinDiff in collected trot. These were thought to reflect left–right differences in muscular strength that affected the ability to raise the forehand and lower the haunches.

## 1. Introduction

Equestrian sports are based on locomotor skills that require varying combinations of speed, endurance, and power. Locomotion is a result of ground reaction forces (GRFs) generated when the hooves press against the ground. The resultant GRF can be represented by three perpendicular components, each having a different effect on the horse’s center of mass (CoM): the vertical component pushes the body upwards, the longitudinal component controls craniocaudal accelerations, and the transverse component is necessary for turning. In a lame horse, the generation of vertical GRF and impulse during the stance phase of the lame limb are reduced compared with the compensating limb [1,2,3,4]. This results in asymmetrical vertical excursions of the CoM and axial body segments on the two diagonals [1,2,3,4].

Inertial measurement units (IMUs) measure accelerations from which displacements are calculated. When attached to specific body segments, they offer an accurate method of determining segmental displacements and rotations. In relation to the study reported here, IMUs characterize body motion [5] and detect left–right asymmetries in the vertical motion of axial body segments and the longitudinal motion of the limb segments [2,6]. These asymmetries are consequences of differences in GRFs generated by the left and right limbs. Forelimb asymmetries mostly affect the poll and withers, while hind limb asymmetries have more effect on the pelvis. The ability of IMUs to detect asymmetries that fall below the threshold of detection of the human eye is particularly useful in the diagnosis of lameness in clinical practice [7].

Interestingly, the use of IMUs has revealed high prevalence of movement asymmetries in various populations of horses that are in active training and are described as “sound” by their owners [3,8,9,10,11]. Similar asymmetries have been reported in horses showing limb length discrepancy [12], trotting around a circle [6], or being ridden at a rising trot [13]. Limited information is available regarding the effect of a rider, per se, on gait symmetry at the trot and, thus, differences in horses’ kinematics at the in-hand evaluation compared with the performance in the competition arena.

A question that needs to be addressed in horses with asymmetrical movements of the axial body segments is whether the horse is lame/in pain or whether other confounding influences are involved. One possible confounder is motor laterality originating in the cerebral cortex and resulting in asymmetrical muscular strength or use of the contralateral limb pairs. There is, as yet, insufficient evidence to support or refute an effect of motor laterality on gait symmetry in trotting horses. One study found that 12 of 13 values that were above the commonly used asymmetry limits for PMinDiff were towards the right side, but no other parameter had the same skewed distribution [14]. A different study of 65 young warmblood horses did not find convincing evidence that vertical movement asymmetry was associated with the horses’ perceived laterality patterns [15].

The study reported here addresses gait asymmetry in high-level, actively competing dressage horses under two practical conditions that occur during international dressage competitions (CDI): trot in hand and ridden trot at slow (collected) and fast (extended) speeds. Trot in hand is part of a mandatory, subjective evaluation of the horse’s health and soundness. It is usually performed on a track at least 30 m long with a firm, level, clean, and non-slippery surface. Horses are led from the left side on a loose rein wearing a bridle or halter. The horse walks away from the inspector for a short distance, trots to the end of the track, turns by walking in a clockwise direction, and trots back to the starting point. A decision is made that the horse is accepted (fit to compete), not accepted (unfit to compete), or questionable, in which case the horse is moved to a holding box for further evaluation. Since there is no appeal against the ground jury’s decision, it is important for the inspection to be fair and honest.

The dressage test is performed on an arena surface that is predominantly sand, to which the addition of other materials is allowed. The prescribed test movements are judged and scored subjectively. Criteria evaluated by judges include left–right symmetry in spatiotemporal variables and spinal movements, and one of the responsibilities of the chief judge is to stop the performance if the horse appears to be lame. The extent to which the horse’s own kinematic pattern and the presence of a rider affect locomotor asymmetries is highly relevant in this context. Lower-level competitions do not have a fitness-to-compete evaluation, which puts the onus on the judge to recognize and assess the importance of locomotor asymmetries.

This study evaluates the vertical ROM (range of motion) and movement symmetry in axial body segments and pro-/retraction of the limbs in a group of experienced dressage horses under conditions simulating the CDI fitness-to-compete inspection and the competition performance. The objectives are to measure and compare locomotor asymmetries under the two conditions and, in particular, to evaluate the effects due to the presence of a rider, the trotting speed of the horse, and the type of footing. The hypotheses are that the vertical ROM of the axial body segments, the asymmetries in minimal and maximal heights of axial body segments, and the asymmetries in limb pro-/retraction at trot are greater when horses are ridden compared with trotting in hand.

## 2. Materials and Methods

### 2.1. Horses and Riders

The subjects were 19 dressage horses (mean ± SD; height: 166.7 ± 7.4 cm; age: 10.7 ± 3.2 years; sex: 5 stallions, 11 geldings, and 3 mares) ridden by their regular riders. Inclusion criteria were that horses were assessed as sound by their trainers and two clinicians, horses and riders were able to perform at Prix St. George level or higher, and they were actively competing in high-level dressage competitions. Prior to acceptance into the study, two experienced lameness clinicians (M.R. and E.H.) performed a clinical examination consisting of visual inspection and palpation of the musculoskeletal system.

### 2.2. Study Design and Data Collection

Data were collected by using 15 wireless ProMove-mini IMUs (Inertia Technology B.V., Enschede, The Netherlands), each of which weighed 20 g. The sensors have two aligned accelerometers that provide a single fused signal with high precision and range, a gyroscope that measures angular velocity in a range of ±2000^o^/s, and a compass to measure magnetic field intensity. The IMUs are actively time-synchronized within a precision of 100 ns and a sampling frequency of 200 Hz, which is ample for trotting data [16]. They transmit over a distance up to 30 m to the Inertia Gateway, which coordinates the individual nodes and streams data through the gateway to a laptop computer running Equimoves software (version 0.0.211001). Additionally, each sensor has an on-board SD card with 2 Gb memory, which stores data if the horse moves outside of the wireless transmission range. Stored data are retrieved on completion of the data collection. For further details, see Bosch et al. [17]. The IMUs were also synchronized with 3 video cameras.

**Figure 1:**
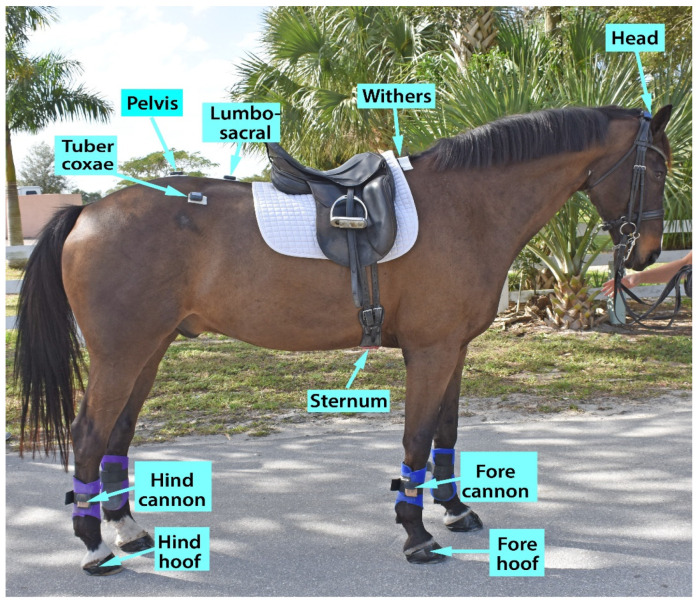
IMU locations. Sensors on the limbs are attached bilaterally. Photo courtesy of Dr. Hilary M. Clayton.

IMU nodes were attached to the poll, withers, lumbar spine region just behind the saddle, left and right tuber coxae, pelvis above and between the tubera sacrale, the cannon region of each limb, and the four hooves. The head sensor was mounted on the crown piece of the bridle by using hook and loop tape. At the withers, lumbosacral region, pelvis, and tuber coxae, sensors were attached to the skin with animal polster and double-sided tape. A lightweight protection boot with a sensor pocket on its lateral aspect was attached to the cannon region of each limb. Hoof sensors were attached on the lateral aspect of the hooves and wrapped with duct tape (Figure 1).

The in-hand evaluation involved walking and trotting the horses approximately 30 m in a straight line over a firm surface. Since horses were evaluated at different venues, the surfaces were not identical and consisted of packed clay, packed gravel, or asphalt. Video recordings made from the cranial, caudal, and lateral views were synchronized with data from the IMUs.

Each horse wore its usual tack for the ridden part of the study. They were ridden in arenas with similar but not identical footing based on sand with added geotextiles/fibers. Horses warmed up in the arena at the rider’s discretion and then performed a dressage test written for the study. It included all dressage gaits performed in straight lines and, when appropriate, on left and right circles, and lateral movements. Two 5 min rest intervals were included. The riders familiarized themselves with the test beforehand, and during testing, it was read to them through ear buds.

### 2.3. Data Processing and Analysis

Data from the sensors were analyzed with EquiMoves software, which converted the recorded vertical accelerations into vertical displacements. Stride segmentation was based on angular velocity data from a gyroscope [17], and stride durations were measured. The cannon-mounted IMUs detected hoof-on and hoof-off events, which marked the transitions between swing and stance phases for each limb [17]. Speed could not be measured because the roof of the covered arenas interfered with satellite communication.

**Figure 2:**
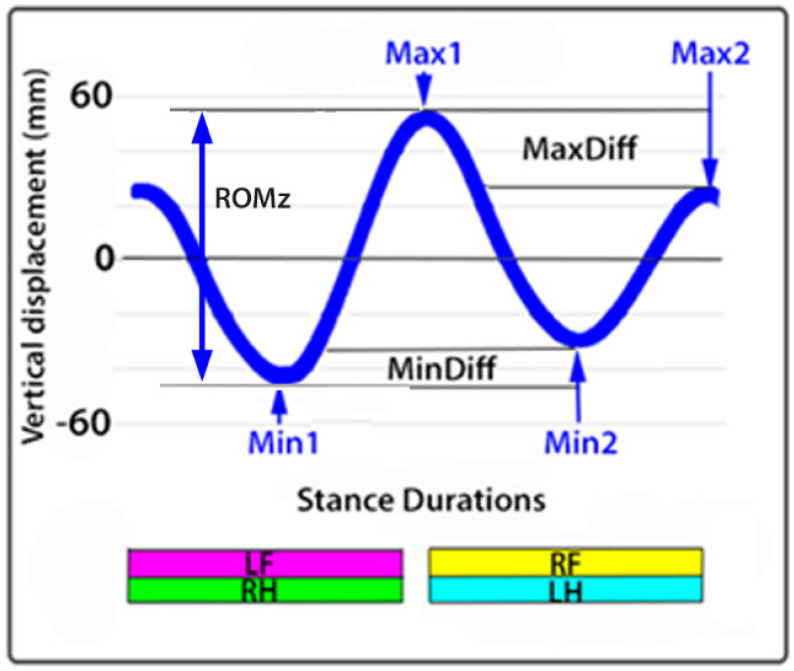
Sinusoidal pattern of the vertical motion of a dorsal midline sensor during one stride at trot showing the two minima, two maxima, and measurement of MinDiff, MaxDiff, and ROMz. The bars at the bottom indicate stance phase durations of the diagonal limb pairs. LF: left fore; RF: right fore; LH: left hind; RH: right hind.

The dorsal midline sensors follow a sinusoidal path with two cycles per stride (Figure 2). The following variables were extracted from the raw data:

- Stride duration: Time elapsing between successive occurrences of the same event in successive strides.
- HROMz, WROMz, and PROMz: Vertical range of motion (ROMz) calculated as the difference between the minimal and maximal heights of the head (H), withers (W), and pelvis (P) sensors during each diagonal stance phase.
- HMinDiff, WMinDiff, and PMinDiff: Absolute difference between the two minima of the heights of the head (H), withers (W), and pelvis (P) sensors during the stride.
- HMaxDiff, WMaxDiff, and PMaxDiff: Absolute difference between the two maxima of the heights of the head (H), withers (W), and pelvis (P) sensors during the stride.
- ProMaxDiff: Difference in maximal protraction between contralateral limbs in late swing. Measured as the absolute difference between left and right cannon segment angles relative to the vertical at maximal protraction when the distal cannon bone is maximally dorsal to its proximal end.
- RetMaxDiff: Difference in maximal retraction between contralateral limbs shortly after lift-off. Measured as the absolute difference between the left and right cannon segment angles relative to the vertical at maximal retraction when the distal cannon segment is maximally palmar/plantar to its proximal end.

### 2.4. Statistical Analysis

Descriptive statistics were performed, and boxplots, showing means, inter-quartile ranges, and individual data points, were created to demonstrate stride duration, ROM, upper-body symmetry parameters, and symmetry in maximum cannon protraction and retraction for the three trot conditions (extended, collected, and in hand).

All variables except stride duration were square root-transformed to achieve a normal distribution of model residuals. By using R-studio (version 3.6.3) and the package lme4 (version 1.1), linear mixed models were obtained for all variables with horse as a random effect and trot conditions (in hand, extended, and collected) as fixed effects. For pairwise comparisons, *p*-values of ≤0.05 were regarded as significant.

## 3. Results

All horses completed all phases of the data collection required for this study. The following description refers only to statistically different findings (*p* ≤ 0.05).

### 3.1. Stride Duration

**Figure 3:**
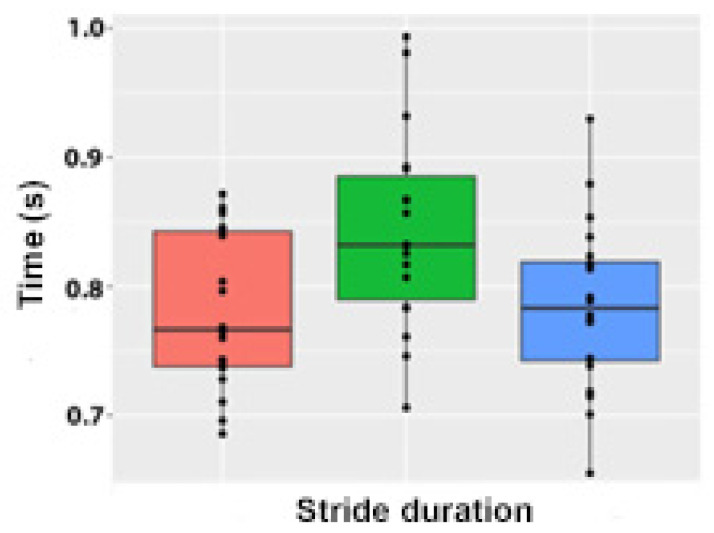
Boxplot showing arithmetic means, inter-quartile ranges, and individual data points for stride duration during extended (orange), collected (green), and in-hand (blue) trot.

Stride duration (LSmean) was significantly longer in collected trot (0.85 s) compared with trot in hand (0.78 s) and extended trot (0.78 s), which did not differ from each other (Figure 3).

### 3.2. Vertical Range of Motion

**Figure 4:**
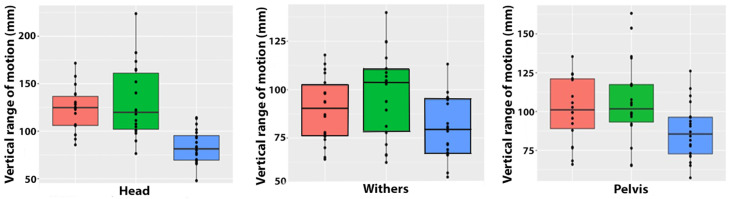
Boxplots showing arithmetic means, inter-quartile ranges, and individual data points for vertical range of motion of the head (**left**), withers (**center**), and pelvis (**right**) for extended trot (orange), collected trot (green), and in-hand trot (blue).

Differences between the vertical ROM in the three types of trot followed the same pattern for the head, withers, and pelvis, with trot in hand having a significantly smaller vertical ROM than both ridden conditions, which did not differ from each other (Table 1, Figure 4).

### 3.3. MinDiff

**Figure 5:**
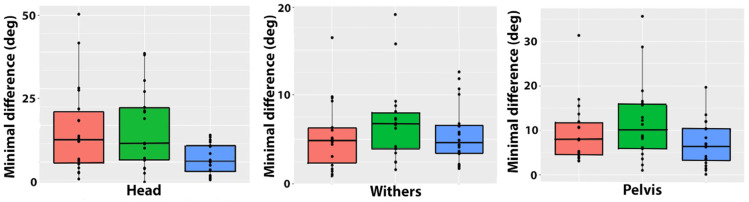
Boxplots showing absolute left–right differences in minimal values for height of the head (**left**), withers (**center**), and pelvis (**right**) for extended trot (orange), collected trot (green), and in-hand trot (blue).

MinDiff values for the head, withers, and pelvis are shown in Table 2 and Figure 5. HMinDiff was larger for both types of ridden trot than trot in hand. WMinDiff had low values that did not differ among trot conditions. PMinDiff was higher for collected trot than trot in hand.

### 3.4. MaxDiff

**Figure 6:**
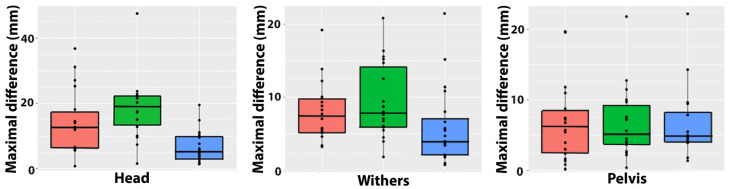
Boxplots showing absolute left–right differences in maximal values for the height of the head (**left**), withers (**center**), and pelvis (**right**) for extended trot (orange), collected trot (green), and in-hand trot (blue).

MaxDiff values for the head, withers, and pelvis are shown in Table 2 and Figure 6. HMaxDiff and WMaxDiff were higher in both types of ridden trot compared with trot in hand, but there were no differences between extended and collected trot. There were no significant differences in PMaxDiff among trot conditions.

### 3.5. Limb Protraction and Retraction

**Figure 7:**
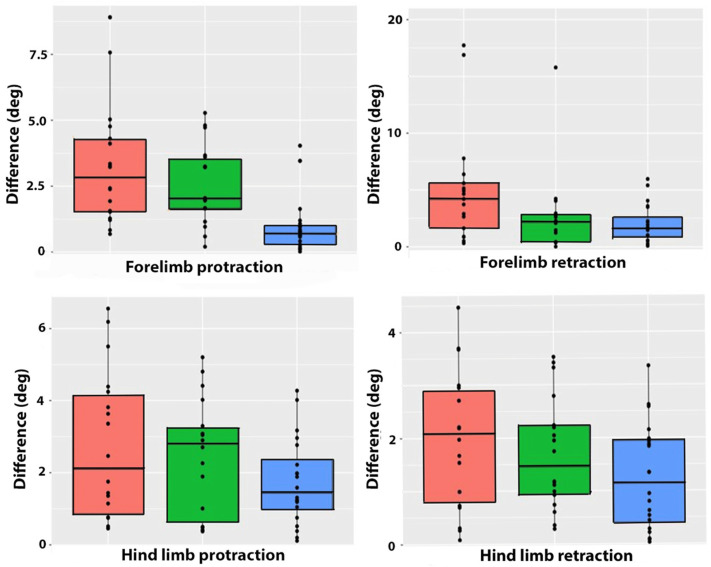
Boxplots showing arithmetic means, inter-quartile ranges, and individual data points for absolute left–right differences in contralateral limb maximal protraction (**left graphs**) and contralateral limb maximal retraction (**right graphs**) in the forelimbs (**top row**) and hind limbs (**bottom row**) for extended trot (orange), collected trot (green), and in-hand trot (blue).

**Figure 8:**
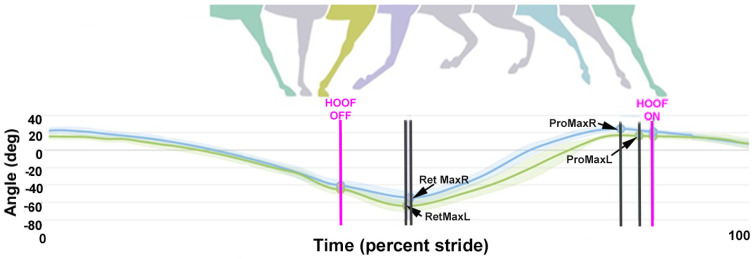
An example from one horse of the magnitude and timing of maximal protraction and retraction in the left (green) and right (blue) forelimbs. Illustration courtesy of Rosalie Bos.

**Figure 9:**
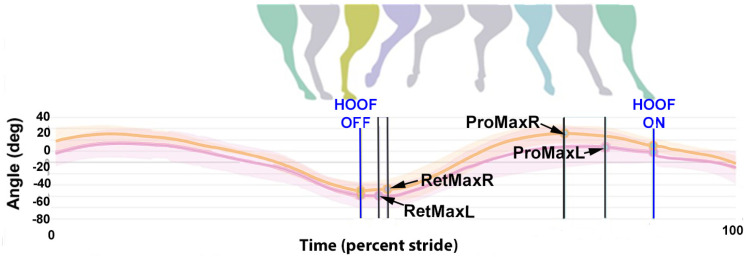
An example from one horse of the magnitude and timing of maximal protraction and retraction in the left (pink) and right (orange) hind limbs. Illustration courtesy of Rosalie Bos.

Fore ProMaxDiff for the left and right limbs in late swing (Table 3, Figure 7) did not differ between collected and extended trot, but both ridden conditions had a larger asymmetry in the forelimb protraction angle than trot in hand. Fore RetMaxDiff did not differ among trot conditions. In the hind limbs, ProMaxDiff was higher in extended trot than collected trot or trot in hand, which did not differ from each other. HindRetMaxDiff did not differ among the three trot conditions (Table 3, Figure 8 and Figure 9).

## 4. Discussion

This study compared stride variables of actively competing dressage horses trotting in hand on a firm surface and being ridden at collected and extended trot on a soft arena surface. This mimics the situations during the fitness-to-compete evaluation and the dressage test performance, respectively, at CDI competitions. The fit-to-compete examination involves visual evaluation by members of the ground jury in consultation with an FEI official veterinarian. The horse should appear healthy and free from marked gait abnormality or lameness at the trot. Horses that are judged to be lame are excluded from the competition.

Issues influencing the visual examination include the limited temporal and spatial resolution of the human eye [18], the lack of agreement even between experienced lameness clinicians [19], the possibility of expectation bias in which an individual’s judgement is influenced by past events, and a perception among competitors that subjectivity may influence the outcome. The possibility of using an objective method to detect gait asymmetries indicative of lameness has been raised.

Asymmetry is a hallmark of lameness but is also a feature of normal gait in people [20] and likely also in animals. Small but consistent asymmetries in the GRFs generated by the left and right legs result in asymmetrical vertical oscillations of the CoM [21]. Small differences between the left and right sides of the body in spatiotemporal variables describing the magnitude or timing of the movements or forces may be regarded as functional rather than pathological asymmetries [8,11]. The human eye may not have sufficient temporal or spatial resolution to detect subtler differences, which is one of the benefits of using IMUs. With regard to lameness detection, the trained human eye has shown better consistency within than between individuals, but IMUs perform better in detecting subtle asymmetries and are more consistent than humans.

Non-pathological gait asymmetries become important when evaluating horses on an individual basis, as in a pre-purchase examination, lameness evaluation, or determination of fitness to compete. Contralateral asymmetries in the vertical trajectory of axial body segments can be detected by IMUs attached to the head, withers, and pelvis. A more difficult problem is knowing whether the asymmetries are simply individual variations of normality or manifestations of pathology. In other words, the challenge lies in defining the limits of normality and determining whether there is overlap between the degree of asymmetry under normal and pathological conditions. Considerable information has been gathered from studies of horses of different ages, breeds, sport disciplines, and levels of training [8,14,15,22,23,24,25,26,27,28,29], confirming that the limits of asymmetry vary among different populations of horses. Therefore, specific limits of asymmetry would need to be established for each sport before objective gait symmetry measurements could be used to determine fitness to compete.

The established method of in-hand fit-to-compete inspections is being challenged in some countries. It has been suggested, for example, that horses could be evaluated while being ridden during competition warm-up. In addition to the challenges inherent in evaluating horses in hand, asymmetry during a ridden evaluation is affected by the rider–horse weight ratio [30], the rider’s posture [31], the rider’s asymmetry pattern [32], the fit of the saddle [33], whether the rider is posting or sitting in the saddle [13], and the influence of the rider’s aids on the horse’s performance as presented in the results shown here. Evaluation of the horse in hand avoids the many confounding factors associated with the tack and the rider.

The use of two different surfaces was an integral part of the study design. The footing for the fitness-to-compete evaluation is firmer than the competition footing, which implies higher concussive forces during impact, higher peak loading forces at midstance, and greater rotational shear resistance during breakover [1,34]. On a firm surface, the hoof is decelerated abruptly after contact, which exacerbates discomfort, for example, in horses with bone or joint problems, such as arthritis. During push-off, the toe cannot penetrate a firm surface, resulting in high tensile forces in the distal check ligament and deep digital flexor tendon, which exert pressure on the navicular bone and its bursa. Thus, the fit-to-compete evaluation on a firm surface is likely to exaggerate gait asymmetries associated with lameness and facilitate the detection of mildly lame horses prior to the start of competition. In contrast, the footing in the competition arena is formulated to dampen impact accelerations more gradually and provide appropriate shear resistance to allow for toe penetration while still providing stability during push-off [34]. A softer surface tends to increase stride duration and between-measurement variation [35], but only collected trot showed a longer stride duration in the study reported here. This is consistent with a previous study showing that collected trot has a longer stride duration than extended trot [36], so this is regarded as a gait-related change. The fact that we found no differences in symmetry parameters between hard and soft surfaces agrees with Marunova et al. [37], who reported no difference in head or pelvic symmetry parameters in horses trotting on hard vs. soft surfaces.

Ideally, dressage horses change speed within a gait by increasing stride length while maintaining a consistent stride duration/rate. A visible change in the stride rate is penalized. A previous study in a comparable group of horses ridden in a sand arena had significantly longer stride duration in collected trot (0.78 ms) than extended trot (0.72 ms), which was associated with large reductions in stance duration in both fore- and hind limbs during the extensions [36]. Those values are somewhat shorter than the 0.85 ms and 0.78 ms reported here and may be related to the differences between a sand surface versus a composite surface.

With regard to the rider’s influence on the horse’s movement, effects can be categorized as gravitational and inertial changes due to the rider’s weight and movements vs. trained responses to the rider’s aids. When a rider sits passively, peak vertical GRF increases in both the fore- and hind limbs of the horse [38]. The presence of a rider can change the vertical motion symmetry of the horse’s head and pelvis, but the effects vary among individual horses [30]. Several studies have evaluated how rider weight, expressed relative to the weight of the horse, affects the horse’s movement. When very heavy riders performed rising trot, locomotor asymmetry increased to the extent that the horses appeared temporarily lame [30]. Rising trot, per se, induces locomotor asymmetry, because when the rider pushes against the stirrups during the rising phase, it creates downward momentum, which counteracts hind limb push-off and simulates push-off lameness in the hind limb the rider sits on [13]. In the study reported here, all riders were of an appropriate size and weight for their horses, and they rode in sitting trot throughout. In CDI competitions, all trot work is performed sitting, so assuming the riders’ weight distribution is symmetrical, the rider’s weight has an equal effect on the two diagonals [14].

Asymmetries in muscular strength are associated with asymmetrical GRF generation on the left and right sides of the body in people with no self-reported injuries or pain. And they result in consistent asymmetry in CoM vertical oscillations [21]. One leg is described as having a propulsive function characterized by generating more hip power at push-off, while the other leg plays a greater role in support associated with power absorption at the knee [20]. If laterality plays a similar role in horses, it may offer an explanation for some non-pathological asymmetries in the movements of the axial body segments.

Head movements are more susceptible to external influences and distractions than those of the withers or pelvis [39]. In sound horses, asymmetries related to distractions are somewhat random and can be removed mathematically by a signal decomposition method [40]. Another factor with the potential to influence head height variables is neck position. Unridden horses choose to walk with the neck almost horizontal, but in trot, the neck is raised, and the head is carried about 20 cm higher than at walk [41]. The elevation of the neck relieves tension from the nuchal ligament and other elastic structures in the dorsal neck, so the head oscillation pattern becomes more dependent on active muscular contractions involving mainly the splenius and cervical trapezius [42]. This effect is amplified in highly trained dressage horses, which adopt an even higher head and neck posture, implying greater reliance on muscular support and the possibility of contralateral, strength-related asymmetries being responsible for the larger HMinDiff and HMaxDiff in ridden horses.

Several studies have applied limits of symmetry based on having one or multiple asymmetry parameters >6 mm for HMinDiff or HMaxDiff or >3 mm for PMinDiff or PMaxDiff, with the SD less than the mean value. Vertical motion exceeding these values has been reported in 83% of Standardbred foals and 45% of Swedish warmblood foals [25]. Asymmetries are even more prevalent in Standardbred yearlings, with 93% exceeding these limits during in-hand trials and 94% during track trials. Interestingly, 20% of horses switched sides between in-hand and track trials. There was no group-level effect between in-hand and track trials, but there was considerable individual variation [25]. The high prevalence of vertical motion asymmetries in such young horses suggests that they are non-pathological. Based on these limits, a large number of experienced performance horses have been classified as asymmetrical. As an example, one or more values fell outside the limits of symmetry in horses trotting in a straight line on hard/soft surfaces, in 67/74% of dressage horses, 67/75% of eventers, and 72/66% of show jumpers [14].

The fact that the horses’ axial body segments showed a larger range of vertical motion with a rider supports the first part of the experimental hypothesis that vertical excursions are greater in ridden horses compared with unridden horses. The higher ROMz values for horses ridden at sitting trot imply greater energy expenditure to project the combined bodyweights of horse and rider more vertically, which is contrary to the natural tendency of the neuromotor control system to conserve energy, as shown by the lower values for ROMz when trotting in hand. The sport of dressage rewards competitors for moving with energy and impulsion, which implies the generation of large vertical GRFs to propel the horse’s CoM into a higher trajectory during the suspension phases [43]. The larger vertical excursions of the head, withers, and pelvis in ridden horses are interpreted as an effect of the rider’s aids encouraging the horse to move with greater impulsion.

The emphasis on having large dorsoventral displacement of the CoM during trotting [44], favors the selection of dressage horses with the ability to project their body into lofty suspensions. Horses with larger overall vertical excursions of the CoM are likely to show larger absolute differences in height between the left and right minima and maxima of the axial body segments than horses with a smaller range of vertical motion. Sport-specific requirements for energy efficiency vs. extravagant movements are reflected in the different threshold levels reported for lameness detection in horses competing in different sports when evaluated by using the same measurement system [23,45]. To compensate for differences in the height of the withers of dressage horses, the evaluation of relative rather than absolute threshold differences is used to normalize for inherent height-related differences in ROMz and its effects on the minima and maxima. This procedure is offered as an option in some gait evaluation systems.

As dressage training progresses, the roles of the fore- and hind limbs become more specialized, with the hind limbs providing more propulsion, while the forelimbs control speed and the direction of movement. Fore- and hind limbs also play different roles in the postural changes associated with developing self-carriage, which is characterized by rotating the body in a nose-up direction around the CoM through a combination of lengthening the forelimbs to raise the withers and shortening the hind limbs to lower the pelvis [46]. As a result of their different mechanical responsibilities, forelimb asymmetries affected primarily MaxDiff, and hind limb asymmetries affected primarily MinDiff. These increases in asymmetries in the minimal and maximal heights of the midline markers in ridden horses support the second part of the experimental hypothesis.

During trotting, the hind hoof typically lifts off slightly earlier than the diagonal forehoof, and the CoM moves ahead of the still-grounded forehoof in terminal stance [47]. This allows the forelimb to exert extra push-off force to elevate the forehand into the suspension phase [46]. The horse’s ability to raise the withers is a characteristic of self-carriage, with maximal withers height occurring around the time of forehoof lift-off into the suspension phase [46]. The fact that WMaxDiff was larger for both ridden conditions may reflect differences in neural drive or strength of the extensor musculature between the left and right forelimbs when the rider creates more uphill carriage. This type of asymmetry can simulate forelimb push-off lameness [48].

The pelvis, on the other hand, showed asymmetry in PMinDiff, which was greater in collected trot vs. trot in hand. The role of the hind limbs in collection is to lower the haunches by accepting weight with the joints in a more flexed position. This is controlled by eccentric contractions of the extensor musculature, primarily the gluteals and hamstrings. Of the three conditions evaluated, collected trot would be expected to show the greatest lowering of the haunches and require the greatest eccentric muscular strength. In lame horses, an increase in PMinDiff would be ascribed to a weight-bearing type of lameness, since the lowering of the pelvis is correlated with peak vertical GRF [48]. In dressage horses, asymmetrical neural drive or strength in the gluteal and hamstring muscles could simulate a weight-bearing hind limb lameness. It has been reported that when horses are ridden by a dressage rider [39] or are ridden with greater collection [37], hind limb asymmetry/lameness increases.

Normal human gait has been reported to show clear asymmetries in spatiotemporal variables during swing, including foot position, which are regarded as a normal expression of laterality so long as they do not exceed 8-10% [49]. Asymmetries in plantar-flexor electromyographic activity have also been related to limb dominance, and it is possible that they are similarly related to the effects of laterality in horses. Asymmetries in limb pro- and retraction have been associated with lameness [3,50,51]. For example, in forelimb lameness, the lame limb tends to be less protracted and, in general, to have a more vertical orientation throughout stance, which allows it to support the body in an elevated position. The body is then lowered during the stance phase of the compensating limb, which has higher vertical GRF and rotates through a greater range of angular motion during stance [52].

Dressage riders encourage their horses to perform with greater cadence and expression of the forelimbs, which includes showing greater forelimb protraction in the swing phase. Some horses have a visible difference between the left and right forelimbs in their positions of maximal protraction which would be recognized as higher ForeProDiff for the ridden conditions as reported here. Asymmetrical contributions of the forelimb musculature to limb elevation and protraction may be responsible for the larger asymmetry in horses being ridden to show greater expression compared with those trotting in hand. The hypothesis regarding larger asymmetries in limb pro-/retraction in ridden horses was supported by larger differences in forelimb protraction when horses were ridden at collected and extended trot and for hind limb protraction in extended trot only. The data indicating there were no significant differences among the conditions in the maximal retraction angles of the fore- or hind limbs do not support our hypothesis.

There is considerable variability in assessments of gait symmetry or lameness among owners, veterinarians, and objective measurement systems [11]. Subjective evaluation is inconsistent, even between experts [11,12], and it seems likely that fitness-to-compete assessments may be equally inconsistent. The human eye does not recognize asymmetry until the difference reaches at least 25% [18], whereas IMUs detect subtle differences below the detection threshold of the human eye [7]. The inherent objectivity and accuracy of evaluation using IMUs at the fitness-to-compete evaluation would be fair to competitors and provide an equal playing field if appropriate threshold values that are specific to this population of horses could be established [23,45]. However, it is important that the IMUs are precisely placed over the underlying anatomical landmarks and correctly oriented [53,54,55], especially when sensors are applied by different individuals.

Markerless tracking has shown results comparable to IMUs for the classification of asymmetries under field conditions [56,57]. Thus, data collection at the fit-to-compete evaluation could be collected with minimal adaptation of the current procedure and without concerns about the precision of marker attachment. However, defining acceptable limits of asymmetry for trot in hand presents the same problems as marker-based evaluations. In both cases, the large variability and presence of numerous outliers is a significant issue when screening individuals.

Limitations to the study include the relatively small number of participants; a much larger pool of mature, experienced competitors would be needed to establish the limits of asymmetry in this population. Horses were evaluated on different surfaces for in-hand and ridden data collection, and data were collected at different venues to mimic competition conditions, so these were not regarded as limitations. All horses were specialized in the same sport and were of similar ages and levels of training, which is required for a single-sport evaluation, but the results should not be generalized to other equine populations.

## 5. Conclusions

The mechanical effects of the rider’s weight interact with trained responses to the rider’s aids to change the dressage horse’s posture and alter the functional responsibilities of the fore- and hind limbs. The higher ROMz of all axial body segments when horses were being ridden compared with trotting in hand resulted from experienced dressage horses developing greater impulsion in response to the rider’s aids. Increases in WMaxDiff, PMinDiff, and Fore ProMaxDiff were ascribed to the riders’ influence on impulsion, collection, and forelimb expression, respectively, with the contralateral limbs responding to different degrees, perhaps as a consequence of asymmetrical muscular strength. The differences presented here constitute a step towards establishing normative asymmetry values for a population of trained dressage horses. This is relevant to discussions about the use of objective tests or ridden evaluations to determine whether a horse is fit to compete.

## Figures and Tables

**Table 1:** Linear mixed model output with pairwise comparison for vertical ranges of motion among trot conditions. Values are LSmeans with 95% confidence intervals for ROM (mm). For each sensor location (head, withers, and pelvis), LSmean values with different superscripts differ significantly (*p* < 0.05).

Sensor Location	Condition	LSmean	Lower C.I.	Upper C.I.
Head	Extended trot	123.4 ^b^	107.8	140.1
	Collected trot	129.3 ^b^	113.3	146.4
	Trot in hand	82.4 ^a^	70.2	95.5
Withers	Extended trot	90.3 ^b^	79.7	101.5
	Collected trot	97.5 ^b^	86.5	109.1
	Trot in hand	79.3 ^a^	69.6	89.5
Pelvis	Extended trot	102.3 ^b^	89.6	115.9
	Collected trot	106.9 ^b^	93.9	120.8
	Trot in hand	86.2 ^a^	74.8	98.4

**Table 2:** LSmean values from mixed model analysis with 95% confidence intervals for mean absolute values of MinDiff and MaxDiff of head, withers, and pelvis (mm). For each sensor location, LSMean values of MinDiff or MaxDiff with different superscripts differ significantly (*p* < 0.05).

		MinDiff			MaxDiff		
		LSmean	Lower C.I.	Upper C.I.	LSmean	Lower C.I.	Upper C.I.
Head	Extended trot	17.3 ^b^	12.4	23.1	12.8 ^b^	8.2	17.9
	Collected trot	17.5 ^b^	12.6	23.3	16.9 ^b^	11.9	22.8
	Trot in hand	10.3 ^a^	6.7	14.6	5.8 ^a^	3.2	9.1
Withers	Extended trot	4.7 ^a^	2.7	6.8	7.5 ^b^	5.0	10.4
	Collected trot	6.4 ^a^	4.4	8.8	8.6 ^b^	6.0	11.8
	Trot in hand	5.2 ^a^	3.4	7.3	4.9 ^a^	3.0	7.2
Pelvis	Extended trot	8.51 ^a,b^	5.2	12.7	5.7 ^a^	3.3	8.6
	Collected trot	10.5 ^b^	6.7	15.0	5.8 ^a^	3.5	8.8
	Trot in hand	5.9 ^a^	3.3	9.2	5.9 ^a^	3.6	8.8

**Table 3:** LSmean from mixed model analysis with 95% confidence intervals for mean absolute values of ProMaxDiff and RetMaxDiff of the fore- and hind limbs. For forelimbs and hind limbs, LSmean values of ProDiff or RetDiff with different superscripts differ significantly (*p* < 0.05).

		ProMaxDiff (deg)			RetMaxDiff (deg)		
		LSmean	Lower C.I.	Upper C.I.	LSmean	Lower C.I.	Upper C.I.
Fore	Extended trot	2.9 ^b^	1.9	4.0	5.0 ^a^	2.8	7.9
	Collected trot	2.3 ^b^	1.5	3.3	0.7 ^a^	3.9	0.81
	Trot in hand	0.7^a^	0.3	1.3	1.7 ^a^	0.6	3.4
Hind	Extended trot	2.3 ^b^	1.4	3.4	1.7 ^a^	1.0	2.5
	Collected trot	2.1 ^a^	1.3	3.2	1.5 ^a^	0.9	2.3
	Trot in hand	1.5 ^a^	0.8	2.4	1.0 ^a^	0.5	1.6

## Data Availability

Data and templates related to this study can be accessed at: https://uclandata.uclan.ac.uk/id/eprint/502 (accessed on 9 January 2025).
